# Antioxidant Activity of Zein Hydrolysates from Zea Species and Their Cytotoxic Effects in a Hepatic Cell Culture

**DOI:** 10.3390/molecules23020312

**Published:** 2018-02-02

**Authors:** Jorge L. Díaz-Gómez, Margarita Ortíz-Martínez, Oscar Aguilar, Silverio García-Lara, Fabiola Castorena-Torres

**Affiliations:** 1 Agri-Foods Unit. Tecnologico de Monterrey, Campus Monterrey, 64890 Nuevo León, Mexico; cratker@gmail.com (J.L.D.-G.); magaortizmtz@gmail.com (M.O.-M.); sgarcialara@itesm.mx (S.G.-L.); 2 Escuela de Ingenieria y Ciencias. Tecnologico de Monterrey, 64890 Nuevo León, Mexico; alex.aguilar@itesm.mx; 3 Escuela de Medicina, Tecnologico de Monterrey, 64710 Nuevo León, Mexico

**Keywords:** peptide, zein, cancer, apoptosis, maize, cytotoxicity

## Abstract

In recent years, food proteins with bioactivity have been studied for cancer treatment. Zein peptides have shown an important set of bioactivities. This work compares the cytotoxic activity of zein hydrolyzed, extracted from four Zea species: teosinte, native, hybrid, and transgenic (Teo, Nat, Hyb, and HT) in a hepatic cell culture. Zein fraction was extracted, quantified, and hydrolyzed. Antioxidant capacity and cytotoxicity assays were performed on HepG2 cells. The levels of expression of caspase 3, 8, and 9 were evaluated in zein-treated cell cultures. *Zea parviglumis* showed the highest zein content (46.0 mg/g) and antioxidant activity (673.40 TE/g) out of all native zeins. Peptides from Hyb and HT showed high antioxidant activity compared to their native counterparts (1055.45 and 724.32 TE/g, respectively). Cytotoxic activity was observed in the cell culture using peptides of the four Zea species; Teo and Nat (IC50: 1781.63 and 1546.23 ng/mL) had no significant difference between them but showed more cytotoxic activity than Hyb and HT (IC50: 1252.25 and 1155.56 ng/mL). Increased expression of caspase 3 was observed in the peptide-treated HepG2 cells (at least two-fold more with respect to the control sample). These data indicate the potential for zein peptides to prevent or treat cancer, possibly by apoptosis induction.

## 1. Introduction

According to the World Health Organization, cancer will be one of the major causes of morbidity and mortality for the next decade [1]. Among the different types of cancer, hepatic cancer is fifth in prevalence, due to its high mortality and high level of relapse [2]. The main risk factors associated with liver cancer are alcohol consumption, mutation of p53 suppressor gene, and the viral infections, hepatitis B and C, which are related to cirrhosis [3]. Hepatic cirrhosis plays an active role in the development of hepatocarcinoma (HC) because it inhibits liver cell regeneration events, leading to different molecular events in hepatocytes [4]. The most important molecular alteration in hepatocyte cells is the shortening of telomeres, which can cause cell death (such as apoptosis) or activation of reparation mechanisms and fusion of chromosomes, leading to the development of neoplastic cells [5]. HC has been related to oxidative stress (OS). This is a process where reactive oxygen species and reactive nitrogen species stimulate the cell, causing DNA and protein damage [6]. Some effects correlated with the presence of OS and HC are increased production of tumor necrosis factor alpha, which activates OS reactions and induces hepatic fibrosis and eventually, cancer [7]; release of apoptotic factors (oncoproteins and tumor suppressor factors) by damaged mitochondria [8]; shortening of telomeres and damage in the DNA of liver cells, leading to cancer cell mutations [6]; and stimulation of the activity of nuclear factor kappa B, which also induces OS in liver cells [9]. It has been reported that several antioxidants target different steps in the OS pathways in cancer liver cells, including resveratrol, vitamin E, metformin, curcuminoids, and L-carnitine, among others [10,11,12,13,14].

Because there is an important need to develop effective prevention for cancer, chemoprevention has emerged as an anti-cancer approach. Chemopreventive agents are expected to be safe, low-cost, and abundant. These agents contain natural compounds, and they are considered to be safer than synthetic compounds, as they are present in typical diets and are widely available and tolerable. In recent years, research efforts to identify food components to treat cancer have increased significantly [15].

*Zea* genera, represented by maize, is one of the most important food crops worldwide. In 2014, its production reached more than 1 billion tonnes, and it is considered the first mass-produced and mass-consumed crop [16]. Currently, chemopreventive properties of some compounds from *Zea mays* have been identified, including phenolic acids [17,18,19,20], anthocyanins [21,22], carotenoids [23], proteins, and peptides [2]. Proteins from Zea and their derivate peptides have emerged as a promising source of bioactive molecules [24]. The composition and abundance of proteins are dependent on a specific germplasm, because there are different species in the genus *Zea*, ranging from ancient maize to *Zea mays*, with different grades of domestication and breeding to native varieties, hybrid varieties, and biotechnology-modified varieties. An important question is whether there are differences in the protein base compositional profile, determined by domestication and improvement processes. However, no studies exist that have comparatively analyzed the nutraceutical and bioactive properties of evolutionary species of this plant.

Zein is a main protein of cereals, and its peptides have been shown to have an important effect against cancer [25]. The protein content of a kernel is composed of four groups: albumins, globulins, prolamins, and glutelins. The first two groups of proteins are found mainly in the germ, while prolamins and glutelins are mostly found in the endosperm [26]. The role of albumins and globulins is to regulate and control the metabolism of grain, while the function of the other two groups is to store the nitrogen needed for seed germination [27]. The protein content of corn grain is composed mainly of prolamins (zein), followed by glutelins, while albumins and globulins exist in smaller amounts. The term “zein” refers to protein members of the prolamins group, which are soluble in alcohol. The main function of the zein is to store the necessary nitrogen in the grain; it is mainly found as protein bodies in the rough endoplasmic reticulum [28] and represents 50% of the total protein content of grain [29]. The four main fractions of these proteins are α, γ, β, and δ. The α-zein represents 80% of prolamins in the grain.

Scientists have studied the antioxidant activity of the bioactive properties of zein and observed higher bioactivity in the peptides derived from this protein when hydrolyzed. [15]. They have also reported proapoptotic activity of the peptides derived from the protein extracted from corn gluten meal on hepatocarcinoma cells [2,25].

This study analyzes and compares zein hydrolysates from the *Zea* species profile, as well as the antioxidant and cytotoxic bioactivity of this protein and the peptides derived from it. This will support research on new nutraceutical peptides for the prevention of chronic degenerative diseases, such as cancer, in the future.

## 2. Results

### 2.1. Protein Content, Determination, and Zein Quantification

Teosinte (Teo) flour contains the highest protein (5–10%) among all the genotypes; the data were calculated by eliminating the kernel caryopsides (Table 1). These results imply that the domestication process has caused a decrease in the total protein content in the kernel. Additionally, the Teo variety contains the highest zein concentration per gram of dry flour, compared with the other maize varieties (native (Nat), hybrid (Hyb), and transgenic (HT), as well as the highest percentage of zein with respect to total protein, with no differences in the Nat and Hyb varieties. Thus, modern maize kernels have lost zein content compared to the ancestral variety. Enzymatic hydrolysis was less effective in the Hyb variety compared with the other maize varieties.

### 2.2. Sodium Dodecyl Sulfate Polyacrylamide Gel Electrophoresis

**Figure 1:**
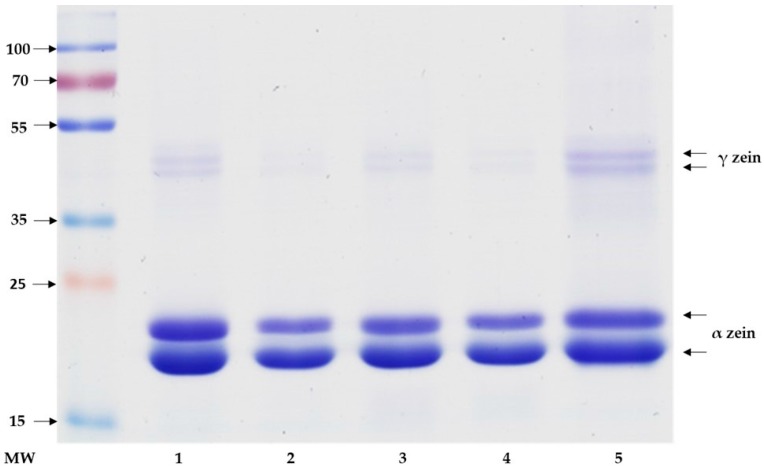
Polyacrylamide gel of the native zein extracts. Protein bands are observed between the molecular weights of 20 and 25 kD in all samples. In addition, two bands of approximately 45 and 48 kD can be seen. (1) Teo, (2) Nat, (3) Hyb, (4) HT, (5) Zein (Sigma-Aldrich, Z3625). MW—molecular weight.

Bands can be observed between 20 and 25 kDa in all samples that correspond to the α-zein fraction in the polyacrylamide gel for zeins (Figure 1). Additionally, bands of approximately 45 and 48 kDa can be observed, corresponding to the γ-zein fraction. Thus, the zein extracts of all the samples present the same protein band patterns and similar weights as the protein used as the standard, so the presence of α- and γ-zeins can be confirmed in the studied genotypes.

### 2.3. Antioxidant Capacity Determination

Antioxidant activity was determined using the oxygen radical absorbance capacity assay. Table 2 shows that the Teo native protein possesses higher antioxidant capacity with respect to the Nat native protein. The zein extract of the Teo variety presented the highest antioxidant capacity (673.40 µM TE/g of zein) compared with the rest of the maize varieties (Table 2). The Hyb variety presented the highest antioxidant capacity of hydrolyzed zein (1055.45 µM TE/g of peptide), while the HT variety presented the lowest activity among all the varieties (742.32 µM TE/g of peptide). Furthermore, all the antioxidant values are higher in the peptides, compared with those of the native zein. Thus, the antioxidant capacity of zein has decreased due to the domestication process of maize. Additionally, the antioxidant capacity is increased considerably with zein hydrolysis, but it does not conserve the differences among the *Zea* varieties previously observed. The Hyb variety presented the highest increase in antioxidant activity after hydrolysis (10-fold more), while Teo presented the lowest (1.5-fold).

### 2.4. Cytotoxicity Assay

A cytotoxic assay was made using whole zein extracts (protein not hydrolyzed), presenting higher half maximal inhibitory concentration (IC_50_) values (data not shown); however, the protein presented problems with solubility in the culture medium and proliferation effects on cells, so this assay was discarded in further analyses. Later, soluble peptides were used for cytotoxic assays (Table 3). At 12 h of treatment with zein peptides, Nat samples presented the highest IC_50_ value, while Teo presented the lowest; significant differences were observed between the zein peptides from Nat and the rest of the samples (Table 3). The average IC_50_ between all samples was 1584 ng/mL. At 24 h of treatment, the Teo and Nat samples were significantly different from Hyb and HT, showing a highest effect by Teo (*p* < 0.05).

### 2.5. Caspase Activity Assay

The activity of caspases 3, 8, and 9 was evaluated to determine whether apoptosis was induced by treatment with zein peptides samples.

**Figure 2:**
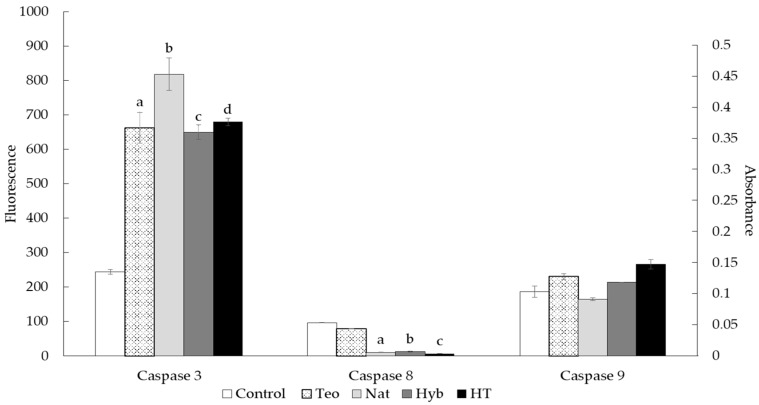
Caspase activity measured in HepG2 cells treated with half-maximal inhibitory concentration (IC_50_) of the peptides obtained from zein of the four *Zea* varieties. Cells left untreated were used as controls. Caspase 3 activity: fluorescence was measured at excitation 485 ± 10 nm and emission 530 ± 12.5 nm at the indicated times. Caspase 8 activity: fluorescence was measured at excitation 400 nm and emission 505 nm. Caspase 9 activity: absorbance was measured at 400 nm. Results are expressed as mean values ± standard deviation (*n* = 4). Treatments that were significantly different are shown with different letters (*p* < 0.05).

As observed in Figure 2, caspase 3 activity was significantly increased in all HepG2 cells treated with zein peptides, compared to controls (untreated cells). Treatment with peptides obtained from Nat presented the most significantly different activity. Caspase 8 showed lower activity than controls. In the case of caspase 9 activity, the treated cells showed a small increase, compared to controls.

## 3. Discussion

### 3.1. Total Protein and Zein Content in Maize Kernels

Concerning the protein content, Berardo et al. [30] and Ignjatovic-Micic et al. [31] reported percentages of protein content within the ranges found in this work. Other authors compared the kernel protein composition between transgenic maize and hybrid varieties, finding a greater protein concentration in the former [32]; we found that the biotech variety had the lowest percentage. Furthermore, by comparing the protein content of teosinte to hybrid varieties, Wang et al. [29] found that the ancestral variety had a higher percentage of protein, which is consistent with the findings presented in this study. This indicates that the evolutionary process of domestication possibly caused a decrease in protein content in maize.

Zein’s main function is to store nitrogen needed for kernel germination. This protein represents about 50% to 70% of whole-kernel proteins [29]. Giuberti et al. [33] reported an average zein concentration of 33 mg/g in dry maize flour, which is lower than the results presented in this study. It is noteworthy to mention that these authors used yellow maize varieties. In contrast, Giuberti et al. [34] reported similar results to ours, but used different solvents for the zein extraction. This difference in solvent utilization could explain the diversity of results of zein extraction in many studies. The results of this study are consistent with the results described by Wang et al. [29], in which teosinte had a higher content of zein, with respect to hybrid and commercial varieties. The content of zein also corresponds to the values obtained in the proximal evaluation of protein, where teosinte presented a higher content. This is because there is a higher proportion (50–70%) of zein than the rest of the grain protein, because it fulfills a structural and storage function [35].

The extracted zein from the different varieties presents the same molecular weights as the main fraction of the reference protein. The α-zein fraction, which can be observed between 20 and 25 kDa, and the γ-zein dimers, between 45 and 48 kDa, correspond with the results reported by Anderson and Lamsal [27] and Giuberti et al. [34]. This confirms the extraction of these fractions in this study. The β- and δ-zein fractions could not be observed in the electrophoresis, because these fractions require reducing conditions to be extracted [26]; also, the extraction method used could obtain α- and γ-zein, and according to Sofi et al. [36], these two fractions constitute the main proportion of the four fractions. α-zein represents 80% of maize kernel prolamins, so it stores the majority of nitrogen necessary for the seed [37]. The γ-zein band intensity is lower than the bands corresponding to the α-zein, because the latter is in greater proportion than to the rest, corresponding to 71% to 85% of zein, according to previously reported information [27].

### 3.2. Antioxidant and Cytotoxic Activity of Zein and Its Peptides

The antioxidant activity of native zein reported in this study was higher in the Hyb and HT samples. It is important to mention that only a few studies have reported the antioxidant activity of native zein and the peptides obtained after enzymatic hydrolysis [38,39]. Additionally, several antioxidant assays have been used to analyze the activity of zein peptides, including the methodology reported in this paper [15,40,41,42,43].

However, comparing the results of the antioxidant activity found in this work with other results reported, it is observed that the antioxidant activity found in zein peptides is higher than that reported by Zhou et al. [44] (28.1–58.15 vs. 814.15–1055.45 µmol Trolox equivalents), and other authors report similar activity to that presented in this work (935.43 and 833.34 µmol Trolox equivalents) [45]. We must clarify that the sizes of the peptide fractions used in the above studies were smaller (<3 kDa). A direct comparison, in this case, must be made with caution, since important differences regarding the starting material (zein vs. corn protein/gluten meal) and hydrolysis conditions could affect the size and sequence of the active peptides.

This is the first time that results of the antioxidant activity of zein teosinte have been reported. These results confirm that enzymatic hydrolysis with alcalase increases antioxidant activity in the peptides, probably due to a wider capacity of the enzyme for protein hydrolysis, since it attacks the peptide bond amide groups, resulting in different peptides not previously observed. Further sequencing or peptide structure determinations should be done. In addition, using a different enzyme or enzyme cocktail could lead to a different peptide profile from the same samples, which has to be isolated and fully characterized.

The proapoptotic activity of peptides extracted from corn gluten meal protein has been reported in a HepG2 cell line [2]. This has led to several investigations on its potential use against cancer. In this study, the zein peptides extracted from the *Zea* varieties have cytotoxic activity, with IC_50_s ranging from 1155.56 to 1781.63 ng/mL at 24 hr. Li et al. [2] reported significant inhibitory activity above 2000 µg/mL. It is important to clarify that Li et al. [2] studied cytotoxic activity without specifying which protein fraction was used to obtain their peptides. Comparing the results of the cytotoxic activity of peptides in this study, we found inhibitory activities below those reported by these authors. It should be considered that the cells were subjected to different incubation times with the peptides in that study. This would explain the different activity reported in the present study. In another study, where peptides derived from rice [46] were analyzed, a lethality rate of 84% was shown at a dose of 1000 µg/mL in HepG2 cells, higher than that reported in this study. Our results contrast with other authors; the lethality in this study was found in lower doses of extracted peptides. Barrio et al. [47] examined the antitumor activity of protein fractions and peptides from amaranth and reported that the native protein had an IC_50_ dose of 1 mg/mL, whereas peptides presented this activity at lower concentrations (0.5 and 0.6 mg/mL) [47]. Therefore, most anticancer activity has been studied in peptides and not in proteins; for example, Jeong et al. [48] found that lunasin extracted from barley grains activated tumor-suppressor genes of the cell cycle.

Hsieh et al. [49] analyzed lunasin extracted from soy and reported antitumorigenic activity, in this case, in mouse fibroblasts exposed to tumorigenic agents [49]. Other studies reported anticancer activity of peptides derived from turnip flour; Xue et al. [50] found inhibitory activity in cell cultures of cervical cancer (HeLa). These authors and Li et al. [2] found an induction of apoptosis of cells exposed to the fraction of peptides derived from seeds; this explains possible mechanisms by which peptides derived from plants have antitumorigenic or anticarcinogenic activity. This indicates that the hydrolysis process enhances the antiproliferative capacity of certain proteins derived from seeds, including zein. Mechanisms that could explain why the peptides extracted from cereal have anticancer activity include induction of apoptosis and inhibition of tumorigenesis by ligand and immune system regulation [24]. Another factor that has been associated with anticancer activity of peptides derived from foods is antioxidant capacity. It is known that oxidative stress causes cell damage that can result in the generation of cancer, so substances with antioxidant peptides could mitigate these negative effects on cell tissues exposed to such stress [51].

### 3.3. Proapoptotic Activity of Zein Peptides

All the zein peptides induced apoptosis in HepG2 cells. Peptides derived from different food sources have been evaluated for anticancer activity and have demonstrated proapoptotic activity by different mechanisms [52,53,54,55], many of which involve activation or inhibition of apoptotic proteins. As mentioned above, previous studies demonstrated that maize peptides have proapoptotic activity. Li et al. reported that maize peptides of low molecular weights increased the expression of apoptotic molecules, like p53 and caspase 3, while Bcl-2 expression (an antiapoptotic factor) was reduced. This correlates with the results presented in this study. Our results show a significant increase in the expression of caspase 3, which is now a point of convergence of the extrinsic and intrinsic pathways of apoptosis [56]; thus, it is a main apoptosis effector. Ortiz-Martinez et al. [25] reported that peptides obtained from maize albumin induced apoptosis in HepG2 cells treated with these peptides; the cells expressed several proteins related to the apoptosis pathway, including pro-caspase 3, the inactivated form of caspase 3 [57]. It seems that maize peptides activate an apoptosis response in cancer cells by inhibiting or activating different proteins, but further study is needed to discover the reason for this activation. The difference in proapoptotic activity between the peptides tested could be explained by the zein of each sample, due to the differences in zein gene expression between maize varieties [58] and in the zein sequence [59]. Analyzing the zein sequence reported elsewhere [60], cysteine and aspartic acid residues are found; if these amino acids are present in the peptides, they could induce an increment of caspase activity, but further studies are needed to clarify this potential.

In contrast, low caspase 8 and caspase 9 activity in HepG2 cells suggests that the extrinsic pathway might not be activated by treatment. Caspase 8 induces apoptosis by activation of death receptors, such as tumor necrosis factor receptor, Fas-associated via death domain, Apo2, and Apo3, among others [48]. Otherwise, caspase 9 can be activated by intracellular stress that activates the mitochondria pathway and is involved the apoptotic intrinsic pathway [47]. However, this does not imply that other apoptotic pathways could be activated. For example, some growth factors can induce apoptosis in cells by activating phosphatidylinositde-3 kinase and v-akt murine thymoma viral oncogene homolog; both molecules regulate the activity of Bad, which is involved in apoptosis via mitochondria [51]. Also, the intrinsic pathway could activate caspase 3 via the activator, p53, which represses some antiapoptotic Bcl2 family proteins and is a cellular inhibitor of apoptosis-1 [52]. Finally, two more proteins can be involved in apoptosis without caspase 8 and 9: endonuclease-G and apoptosis-inducing factor. Endonuclease-G induces nucleosomal fragmentation of DNA if there is an apoptotic stimulus from the mitochondria, and apoptosis-inducing factor translocates to the nuclei, where it initiates chromatin condensation and large-scale DNA fragmentation [53]. Further studies are needed to determine the exact mechanism that zein peptides activate to induce apoptosis.

## 4. Materials and Methods

### 4.1. Germplasm Description

The germplasm was obtained from different sources (Table 4). The teosinte was collected from the central valley of Central Mexico in 2014. The native blue variety was donated by farmers from Chiapas, Mexico. The commercial hybrid, Pioneer 30T83, was bought in Monterrey, Mexico. The Mon-HT-Hercules Plus is a genetically modified maize with an herbicide-resistant trait, kindly donated by Texas A&M University (College Station, TX, USA) for analytic purposes only. Biophysical properties were measured according to Mestres and Matencio [61] and the American Association of Cereal Chemists [62] to verify kernel differences between germplasm accessions.

### 4.2. Flour Sample Preparation

Using a kernel grinder (Krups, Solingen, Germany) 160 g, 6 Hz, 120 mm, 20 g of kernel maize was ground for 50 seconds. The resulting flour was collected in a 50 mL centrifuge tube. The teosinte samples underwent two grinding cycles. The flour obtained from the four varieties was ground once again with a grinder MM400 model (Retsch, Haan, Germany); this process was performed according to García-Lara et al. [63]. Briefly, 5 g of flour of each sample along with a metal pellet were placed in the metallic cylinders of the grinder. Then the cylinders were closed and placed in the grinder, undergoing a grinding process for 4 min. It was necessary to run 2 cycles of this grinding process and to reduce the flour quantity for processing of the teosinte flour.

### 4.3. Zein Extraction and Quantification

Zein was extracted from the flour samples. Ten grams of each sample were defatted with 100 mL of hexane by incubation and agitation at 300 rpm Super-Nuova Multi-Place Stirrer (Thermo Fisher Scientific, Waltham, MA, USA) for 1 h. Next, the supernatant was removed using a pipette and the samples were left in an extraction hood for 24 h. The samples were collected and stored at 4 °C. According to the extraction process by Malumba et al. [64] and Chen et al. [65], with some modifications, 2 g of maize flour of each Zea species, starch (blank) and zein powder (standard, Sigma-Aldrich; St. Louis, MO, USA) were incubated for 30 min with agitation (300 rpm) at room temperature using 20 mL of NaCl 0.5 M, pH 7.0. After this, the mix was centrifuged at 10,000× *g* for 30 min and the supernatant was discarded. Incubation was repeated using 20 mL of Na_2_B_4_O_7_ (0.1 M), pH 10, the samples were centrifuged at 10,000× *g* for 30 min, and the supernatant was discarded. Finally, the third step of incubation, using 20 mL of 95% ethanol was performed, followed by the same centrifugation step (10,000× *g*). Then the supernatant was collected with a pipette and filtered with filter papers (Whatman, Little Chalfont, UK), grade 5. These supernatants were stored at 4 °C.

The kernel protein composition was measured with a near infrared analyzer model DA7250, in accordance with Perten´s methodology (Perten Instruments, Stockholm, Sweden). The Kjeldahl method was performed for the protein determination of teosinte samples. Briefly, a methyl red indicator (1 g of methyl red in 200 mL of ethanol) and a sodium hydroxide solution (450 g of NaOH in 1 L ethanol) were mixed, then filter paper was wrapped with 2 g of potassium sulfate, 0.05 g of cupric sulfate, and 0.105 g of sample; this paper was then placed in a Kjeldahl flask. Subsequently, 2 mL of sulfuric acid was added to each Kjeldahl flask and the samples were digested for 45 min; 10 mL of distilled water was added after the digestion step. Then, each flask was connected to a nitrogen distillation unit along with an Erlenmeyer flask containing 5 mL of boric acid and 20 mL of distilled water. Finally, the NaOH solution was gradually added to the sample to release the nitrogen, and the boric acid solution was titrated with HCl until it turned a salmon-red color. The percentage of protein was calculated using the following formula:

(1) $$\begin{array}{l} \%\;\mathrm{nitrogen}=\frac{\left[\left(\mathrm{mL}\;\mathrm{HCl}\right)\left(N\;\mathrm{HCl}\right)\left(14.007\right)\left(100\right)\right]}{\mathrm{mg}\;\mathrm{of}\;\mathrm{sample}\;\mathrm{weight}}\\ \%\;\mathrm{crude}\;\mathrm{protein}=\left(\%\;\mathrm{nitrogen}\right)\left(6.25\right) \end{array}$$

The results were calculated using the dry weight of each sample and are reported in total percentage adjusted to 100%.

A ninhydrin-based assay was used to quantify the zein obtained from the previous extraction process, with some modifications [66,67]. First, the samples and the standard for the quantification step were hydrolyzed using 360 µL of HCl 6N and 240 µL of each sample at 100 °C for 24 h in a block heater (HACH, CCD Reactor, Loveland, Colorado, USA); then, the tubes were opened and left in the block heater until dry. Once the liquid was evaporated, the samples were suspended with 240 µL of deionized water. Then, a ninhydrin solution was prepared, consisting of 2.5 mL of sodium acetate trihydrate 2N (VWR AnalaR NORMAPUR 27655.260, Radnor, PA, USA), 7.5 mL of ethylene glycol, 200 mg of ninhydrin (Sigma-Aldrich 151173, St. Louis, MO, USA), and 250 µL of stannous chloride suspension (50 mg of SnCl_2_ in 500 µL of ethylene glycol; Sigma-Aldrich 474762 and 324558, respectively). Then, 100 µL of this solution was added to each tube, and they were incubated at 95 °C for 45 min in a thermocycler. Finally, 90 µL of mix reaction from each tube was pipetted to a 96-well microplate, and absorbance was measured in a microplate reader (Biotek Synergy HT, Winooski, VT, USA) at 575 nm. The reading procedure was performed against a blank (deionized water), and a zein standard curve was used to make a curve plot and calculate the zein concentration of each sample.

### 4.4. Zein Hydrolysis

Based on the method described by Bamdad et al. [68] and Zheng et al. [39], with some modifications, new dilutions for each sample were made in a total volume of 10 mL, at a concentration of 1 mg/mL. Then, 5 mL was pipetted and mixed with 4.99 mL of 0.5 M pH 8 solution (phosphate-buffered saline, PBS) and 50 µL of alcalase solution at 0.0525 U/mL. The tubes were heated at 60 °C for 2 h in a block heater (HACH, CCD Reactor). Then, the reaction was stopped by lowering the pH to 7, using HCl 6N. Subsequently, the mix was converted to an ultraconcentrator tube (Spin-X UF 6 5 k Corning 431482; Corning, NY, USA) and centrifuged at 10,000× g for 1 h. The precipitate was collected and stored at 4 °C.

### 4.5. Electrophoresis of Native Zein

Sodium dodecyl sulfate polyacrylamide gel electrophoresis was performed to analyze the zein profile of each extract. An electrophoresis chamber was used (Mini-Protean Tetra Cell Bio-Rad, Hercules, CA, USA) and the polyacrylamide gels were made, in accordance with LaemmLi [69]. Briefly, the separating gel solution was prepared at 12% and converted between chamber glasses. After that, isopropanol was used on top of the gel to ensure proper gel polymerization; the gel was allowed to polymerize for 1 h. The isopropanol was then removed, the stacking gel solution was added on top of the previous 4 gels, and the gel was allowed to polymerize for 1 h. Then, the chamber was filled with running buffer Tris-Glycine-SDS for zein samples Sigma–Aldrich A3574 (Sigma-Aldrich, St. Louis, MO, USA), the gel was placed inside the chamber, and the samples diluted in LaemmLi sample buffer were loaded into the gel (20 µL per well) along with the molecular weight ladder (Thermo Scientific PageRuler Plus; Waltham, MA, USA). All polyacrylamide gels were standardized with a simple concentration of 1 mg/mL per well. The running conditions were 16 mA for the stacking gel and 24 mA for the separating gel. Immediately after the running step was terminated, the gel was removed from the chamber and washed for 3 cycles of 5 min each. Finally, the gels were stained with blue Coomassie solution (Bio-Rad Bio-Safe Coomassie 1610786, Hercules, CA, USA) for 1 h and washed with distilled water for 30 min. Gel pictures were taken using a scanner at 900 dpi resolution (ImageScanner III, GE, Little Chalfont, UK).

### 4.6. Antioxidant Activity

Antioxidant activity was determined using the oxygen radical absorbance capacity assay. Extracts were evaluated following the method described by Gutiérrez-Uribe et al. [70], with modifications, using a standard of Trolox (Sigma-Aldrich, St. Louis, MO, USA) with fluorescein (Sigma-Aldrich, St. Louis, MO, USA). Peroxyl radicals were generated by adding 2,2′-azobis (2-amidinopropane) dihydrochloride (Sigma-Aldrich, St. Louis, MO, USA), and the fluorescence loss signal was monitored in a microplate reader for 1 h. The absorbances of excitation and emission were set at 485 nm and 538 nm, respectively. The results are expressed as μmol of Trolox equivalents (TE) per gram dry weight.

### 4.7. Cytotoxicity Assay

American Type Culture Collection [71] protocols for maintenance of HepG2 mammalian cells (ATCC HB-8065, Manassas, Virginia, USA) were followed. Under sterile conditions and under a double laminar flow hood, cells were cultured using a mixture of 50% Dulbecco’s Modified Eagle Medium (Sigma-Aldrich D6046, St. Louis, MO, USA) and 50% Minimum Essential Media (Sigma-Aldrich 51411C, St. Louis, MO, USA) with 10% fetal bovine serum (Sigma-Aldrich F6178, St. Louis, MO, USA) and 1% antibiotic; the flasks were incubated at 37 °C with 5% CO_2_. The cells were analyzed under a microscope to check their health (pollution) and growth. When the culture reached confluence, the culture medium was removed, the flask with PBS was washed, and the cells were detached with trypsin (Sigma-Aldrich T4549, St. Louis, MO, USA) to be cultured in new flasks. Quantification of cells was done with trypan blue using the Neubauer chamber of a hemacytometer. Cells were counted 4 times from the chamber and an average was obtained. That number was multiplied by 1 × 10^4^ to obtain the number of cells/mL.

According to the neutral red method [72], a colorimetric cytotoxicity assay was performed. Briefly, HepG2 cells, grown to a concentration of 2 × 10^5^ cells/mL, and controls were pipetted in a 96-well plate to a final volume of 200 µL and incubated at 37 °C for 24 h. Subsequently, the medium was removed and the extracted zein and hydrolysates were pipetted into 5 concentrations (from 750 to 3000 ng/mL) in a culture medium (200 µL final volume). This assay was performed in triplicate and incubated at 37 °C for 24 h. Subsequently, the treatment medium was removed, wells were washed with PBS, and 100 µL of staining medium was added to each well. The staining medium (120 µL of neutral red solution, 40 mg of neutral red in 10 mL of PBS, plus 12 mL culture medium, 50% Minimum Essential Medium and 50% Dulbecco’s Modified Eagle Medium) was prepared, and the microplate and was incubated at 37 °C for 2 h. After washing with PBS and adding 150 µL of destaining solution (10 mL of deionized water, 10 mL of 96% ethanol, and 200 µL of glacial acetic acid), the microplate was stirred for 10 min. Absorbance was read at 540 nm in a microplate reader. Culture medium was used as a blank (control without cells), untreated cells (untreated control) and cells treated with 0.1% sodium dodecyl sulfate were used as positive controls. A dose-response curve was performed to determine the IC_50_.

### 4.8. Caspase Activity Evaluation

The activities of caspase 3, 8, and 9 were evaluated. These caspases are involved in the effectors of extrinsic and intrinsic apoptotic pathways, respectively. We used 3 kits to evaluate caspase expression: caspase 3: EnzChek Caspase-3 Assay Kit (Thermo Fisher Scientific, Waltham, MA, USA); caspase 8: ApoTarget Caspase-8 Fluorometric Protease Assay (Invitrogen, Frederick, MD, USA); and caspase 9: ApoTarget Caspase-9 Colorimetric Protease Assay (Invitrogen).

#### 4.8.1. Caspase 3 Activity Evaluation

Cells were cultured by bringing them to at least 1 × 10^6^ cells per reaction. Cell death was induced by cytotoxicity according to peptide treatment. The samples were evaluated twice and were run in triplicate. Negative and positive controls were included in the same plate. Cells were collected and washed with PBS, suspended in the lysis buffer solution (50 μL solution per sample house or control), and incubated on ice for 30 min. Each sample was then centrifuged at 5000 rpm for 5 min. Subsequently, 50 μL of supernatant from each sample was pipetted into a 96-well plate, and 50 μL of the lysis buffer solution without enzyme was also pipetted as a control. Fifty microliters of working solution (Z-DEVD-AMC substrate) was added to each well of the plate and incubated for 30 min. Fluorescence (excitation/emission ~342/441 nm) was measured with a microplate reader (Biotek Synergy HT, Winooski, VT, USA). The readings were compared to a standard curve of 7-amino-4-methylcoumarin to determine the level of caspase activity.

#### 4.8.2. Caspase 8 Activity Evaluation

Cells were cultured by bringing them to at least a concentration of 1 × 10^6^ cells per reaction. Cellular cell death was induced by cytotoxicity according to peptide treatment. The samples were evaluated twice and were run in triplicate. Negative and positive controls were included in the same plate. Cells were collected and washed with PBS, suspended in the lysis buffer solution (50 μL solution per sample house or control), and incubated on ice for 10 min. Fifty microliters of reaction solution (containing 10 mM dithioreitol) was added to each sample, and 5 μL of isoleucine-glutamic acid-threonine-aspartic acid-trifluromethyl coumarin solution was added, followed by incubation for 2 h at 37 °C. Fluorescence was read in a microplate reader (Biotek Synergy HT, Winooski, VT, USA) at an excitation of 400 nm and an emission of 505 nm.

#### 4.8.3. Caspase 9 Activity Evaluation

Cells were cultured by bringing them to at least 3 × 10^6^ cells per reaction. Cellular death was induced by cytotoxicity according to peptide treatment. The samples were evaluated twice and were run in triplicate. Negative and positive controls were included in the same plate. Cells were collected and washed with PBS, suspended in the lysis buffer solution (50 μL solution per sample house or control), and incubated on ice for 10 min. They were then centrifuged for 1 min at 10,000× *g*. The supernatant from each sample was transferred to a tube and incubated on ice. The protein content of the supernatants was determined by the Bradford method to a concentration of 200 μg of protein per 50 μL of lysis buffer solution. Fifty microliters of reaction solution (containing 10 mM dithioreitol) and 5 μL of leucine-glutamic acid-histidine-aspartic acid-p-nitroaniline solution were added to each sample, followed by incubation for 2 h at 37 °C. Absorbance was read at 400 nm in a microplate reader (Biotek Synergy HT). The readings were compared to the readings of the untreated controls to determine caspase activity.

### 4.9. Statistical Analysis

Each determination was performed in triplicate and is presented as average ± standard deviation. Analysis of variance was performed using SLStat statistical software. Differences among means were compared with Tukey tests at *p* < 0.05.

## 5. Conclusions

In this study, we found that the antioxidant activity of zein increased with enzymatic hydrolysis, and Hyb and HT peptides derived from this hydrolysis demonstrated higher cytotoxic activity in a HepG2 cell culture, with higher bioactivity compared with native peptides and with peptides in other studies. Regardless of the source, all peptides showed similar activity, indicating the potential for enzyme hydrolysis as a bioactivity enhancer. Also, we demonstrated that zein peptides induce apoptosis by increasing the expression of caspase 3. However, it is necessary to determine and purify the peptides, and to conduct bioassays with different cell lines with in vivo models to achieve better comprehension of the proapoptotic activity of these peptides, so their potential for preventive and therapeutic use in cancer diseases can be determined.

## Figures and Tables

**Table 1:** Quantification of protein and native zein in flour from diverse maize germplasm.

Germplasm	Protein	Native Zein	
	(%)	mg/g Dry Flour	% Zein/Total Protein
Teo	18.86 ± 0.94 ^a^	46.00 ± 5.39 ^a^	24.39 ± 2.85 ^a^
Nat	9.27 ± 0.11 ^c^	21.39 ± 1.66 ^b^	23.07 ± 2.07 ^ab^
Hyb	13.13 ± 0.21 ^b^	28.55 ± 1.33 ^b^	21.74 ± 1.17 ^ab^
HT	8.51 ± 0.22 ^d^	14.83 ± 2.55 ^b^	17.43 ± 3.46 ^b^

Teo: teosinte; Nat: native blue Chiapas; Hyb: Pioneer 30T83; HT: Mon-HT Hercules Plus. Results are expressed as mean values ± standard deviation; *n* = 3. Superscript letters signify significantly different results at *p* < 0.05 by Tukey test.

**Table 2:** Antioxidant capacity evaluation of native and hydrolyzed zein.

Germplasm	Native Protein	Hydrolyzed Zein
	(µM TE/g of Zein)	(µM TE/g of Peptide)
Teo	673.40 ± 82.93 ^a^	987.53 ± 2.88 ^b^
Nat	87.86 ± 3.60 ^b^	814.15 ± 5.92 ^c^
Hyb	98.92 ± 1.27 ^b^	1055.45 ± 14.69 ^a^
HT	90.84 ± 1.33 ^b^	724.32 ± 3.26 ^d^

Teo: teosinte; Nat: native blue Chiapas; Hyb: Pioneer 30T83; HT: Mon-HT Hercules Plus. Results are expressed as mean values ± standard deviation; *n* = 3. Superscript letters signify significantly different results (*p* < 0.05).

**Table 3:** Half maximal inhibitory concentration (IC_50_) of zein peptides on the HepG2 cell line at various times of exposure.

Germplasm	IC_50_ (ng/mL)	
	12 h	24 h
Teo	1198.69 ± 14.82 ^dc^	1781.63 ± 100.10 ^a^
Nat	2233.74 ± 100.28 ^a^	1546.23 ± 183.77 ^a^
Hyb	1526.44 ± 29.25 ^b^	1252.25 ± 4.8 ^b^
HT	1377.36 ± 21.09 ^c^	1155.56 ± 33.07 ^b^

Teo—teosinte; Nat—native blue Chiapas; Hyb—Pioneer 30T83; HT—Mon-HT Hercules Plus; IC_50_—half inhibitory concentration. Results are expressed as mean values ± standard deviation; *n* = 3. Superscript letters signify significantly different results (*p* < 0.05). Cells without treatment were used as controls and were taken as having 100% viability; sodium dodecyl sulfate at 0.1% was used as positive control.

**Table 4:** Properties description of *Zea* germplasm used for the zein peptides bioactivity study.

Germplasm	Acronym	Classification *	Ecology	Test Weight kg/hL	1000 Kernel Weight (g)	Endosperm Proportion (%)	Endosperm Texture **
Teosinte (*sub mexicana*)	Teo	Ancestral maize	Highlands	75.35 ± 0.25 ^b^	80.67 ± 1.20 ^c^	41.88 ± 5.35 ^b^	2.09 ± 0.2 ^b^
Azul de Chiapas	Nat	Local land race	Tropical	71.63 ± 0.34 ^c^	375.90 ± 5.70 ^a^	83.81 ± 1.45 ^a^	4.20 ± 0.1 ^a^
Hybrid Pioneer 30T83	Hyb	Conventional hybrid	Tropical	76.38 ± 0.17 ^a^	373.51 ± 3.34 ^a^	86.80 ± 0.65 ^a^	4.34 ± 0.1 ^a^
Hybrid HT Hercules Plus	HT	Transgenic hybrid	Temperate	75.65 ± 0.37 ^b^	286.62 ± 3.98 ^b^	86.39 ± 0.86 ^a^	4.32 ± 0.1 ^a^

* Based on evolutionary scale and breeding process. ** Endosperm texture is subjectively determined by viewing the ratio of soft to hard endosperm on dissected kernels: 1 = totally vitreous or hard, 5 = totally soft or chalky. Superscript letters signify significantly different results at *p* > 0.05 by Tukey test.
